# PPAR-γ Gene Expression in Human Adipose Tissue Is Associated with Weight Loss After Sleeve Gastrectomy

**DOI:** 10.1007/s11605-021-05216-6

**Published:** 2021-12-08

**Authors:** Jorge-Luis Torres, Ricardo Usategui-Martín, Lourdes Hernández-Cosido, Edgar Bernardo, Laura Manzanedo-Bueno, Ignacio Hernández-García, Ana-María Mateos-Díaz, Orlando Rozo, Nuria Matesanz, Daniel Salete-Granado, Antonio-Javier Chamorro, Cristina Carbonell, Marina Garcia-Macia, Rogelio González-Sarmiento, Guadalupe Sabio, Luis Muñoz-Bellvís, Miguel Marcos

**Affiliations:** 1 Department of Internal Medicine, University Hospital of Salamanca-SACYL-IBSAL, Salamanca, Spain; 2 Department of Internal Medicine, Complejo Asistencial de Zamora-SACYL, Zamora, Spain; 3 IOBA, University of Valladolid, Valladolid, Spain; 4 Cooperative Health Network for Research (RETICS), Oftared, National Institute of Health Carlos III, ISCIII, Madrid, Spain; 5 Bariatric Surgery Unit, Department of General and Gastrointestinal Surgery, University Hospital of Salamanca, Salamanca, Spain; 6 University of Salamanca, Salamanca, Spain; 7 Department of Vascular Biology and Inflammation, Fundación Centro Nacional de Investigaciones Cardiovasculares Carlos III, 28029 Madrid, Spain; 8 Department of Preventive Medicine and Public Health, Lozano Blesa University Clinical Hospital of Zaragoza, Zaragoza, Spain; 9 Department of Surgery, Complejo Asistencial de Ávila-SACYL, Ávila, Spain; 10 Institute of Functional Biology and Genomics, University of Salamanca, CSIC, Salamanca, Spain; 11 Centro de Investigación Biomédica en Red Sobre Fragilidad Y Envejecimiento Saludable (CIBERFES), Instituto de Salud Carlos III, Madrid, Spain; 12 Molecular Medicine Unit-IBSAL, University of Salamanca-SACYL-CSIC, Salamanca, Spain; 13 Department of General and Gastrointestinal Surgery, Hospital Universitario de Salamanca, Biomedical Research Institute of Salamanca (IBSAL), Universidad de Salamanca, Salamanca, Spain

**Keywords:** Obesity, PPAR gamma, Bariatric surgery, miR-27

## Abstract

**Background:**

The peroxisome proliferator-activated receptor (PPAR)-γ plays a key role in adipose tissue differentiation and fat metabolism. However, it is unclear which factors may regulate its expression and whether obese patients have changes in adipose tissue expression of PPAR-γor potential regulators such as miR-27. Thus, our aims were to analyze PPAR-γ and miR-27 expression in adipose tissue of obese patients, and to correlate their levels with clinical variables.

**Subjects and Methods.:**

We included 43 morbidly obese subjects who underwent sleeve gastrectomy (31 of them completed 1-year follow-up) and 19 non-obese subjects. mRNA expression of PPAR-γ1 and PPAR-γ2, miR-27a, and miR-27b was measured by qPCR in visceral and subcutaneous adipose tissue. Clinical variables and serum adipokine and hormone levels were correlated with PPAR-γ and miR-27 expression. In addition, a systematic review of the literature regarding PPAR-γ expression in adipose tissue of obese patients was performed.

**Results:**

We found no differences in the expression of PPAR-γ and miR-27 in adipose tissue of obese patients vs. controls. The literature review revealed discrepant results regarding PPAR-γ expression in adipose tissue of obese patients. Of note, we described a significant negative correlation between pre-operative PPAR-γ1 expression in adipose tissue of obese patients and post-operative weight loss, potentially linked with insulin resistance markers.

**Conclusion:**

PPAR-γ1 expression in adipose tissue is associated with weight loss after sleeve gastrectomy and may be used as a biomarker for response to surgery.

**Supplementary Information:**

The online version contains supplementary material available at 10.1007/s11605-021-05216-6.

## Introduction

The pathogenesis of obesity involves the interaction of behavioral, environmental, and genetic factors ^1^, which finally results in increased adipose tissue mass due to both adipocyte hypertrophy and hyperplasia, and adipose tissue dysregulation ^2,3^. Further, obesity is associated with altered adipocyte endocrine signaling, which is a key factor in the pathophysiology of obesity-related complications such as insulin resistance or type 2 diabetes mellitus ^4^. The worldwide prevalence of obesity has reached epidemic proportions, increasing between 1975 and 2018 from 4.8 to 12.8% in adults over 18 years ^5^.

Among the several factors involved in adipose tissue regulation, peroxisome proliferator activated receptor (PPAR)-γ, which is highly expressed in white and brown adipose tissue, is considered the “master regulator” of adipogenesis. This transcription factor is a well-characterized regulator of energy metabolism ^6^ and is clearly involved in the pathophysiology of obesity ^6–8^ and related complications ^9,10^. Previous studies, however, have shown conflicting results regarding PPAR-γ expression in adipose tissue of obese patients. Whereas some studies have shown increased expression of PPAR-γ1, PPAR-γ2, or total PPAR-γ in adipose tissue of obese individuals versus controls ^11–18^, other authors have shown decreased expression of these factors ^19–22^, or no changes whatsoever ^23–28^. Different hypotheses have been tested for these discrepant results, including potential differences related to variables such as gender ^17,29^, the degree of insulin sensitivity ^16,25^, the type of adipose tissue analyzed ^30,31^, or the presence of recent weight loss or gain ^15,32^. Despite these research efforts, the exact role of adipose tissue PPAR-γ gene expression in human obesity remains elusive. In addition, accumulated evidence has shown an important role of several miRNAs in the pathophysiology of obesity, including adipogenesis, insulin resistance, and inflammation. Indeed, recent studies have found that miR-27, mainly miR-27a, regulates the PPAR-γ pathway. This miRNA represses PPAR-γ in human multipotent adipose-derived stem cells ^33^ and inhibits PPAR-γ gene expression at both the mRNA and protein levels ^34^. In this scenario, the main aim of this work was to analyze the expression of PPAR-γ and miR-27 in abdominal and subcutaneous adipose tissue to analyze their potential associations with clinical and metabolic variables of obese patients, including weight loss after surgery. In addition, and due to prior controversial findings, we also performed a systematic review on this topic.

## Subjects and Methods

### Subjects

The study population was composed of 43 morbidly obese subjects who underwent laparoscopic sleeve gastrectomy. The control group consisted of 19 non-obese subjects who underwent elective laparoscopic cholecystectomy. All individuals were operated by the same surgical team. Obese patients were followed up after surgery at 1, 3, 6, and 12 months (31 patients completed follow-up at least 1 year after surgery). Diagnosis of obesity was established according to the World Health Organization (WHO) criteria. Demographic, anthropometric, laboratory, and clinical characteristics such as age, sex, height, weight, hypertension, diabetes mellitus, cardiovascular disease, dyslipidemia, obstructive sleep apnea–hypopnea syndrome (OSAHS), hypothyroidism, prior cancer, fasting plasma glucose, total cholesterol, triglycerides, low-density lipoprotein (LDL) cholesterol, and high-density lipoprotein (HDL) cholesterol were collected for each subject in the study. Individuals were not receiving PPAR-γ agonists (thiazolidinediones). Body mass index (BMI) and body fat percentage were calculated before surgery and percentage of BMI reduction (100 − [BMI after surgery/initial BMI] *100) and percentage of body weight loss ([number of kilogram lost after surgery/initial weight] * 100) were calculated during follow-up. All subjects were Caucasian.

### Ethics Declarations

The experimental protocol was approved by the Ethics Committee of our University Hospital, and it complied fully with the Declaration of Helsinki (2008). All subjects were informed of the purpose of the study and gave written informed consent before inclusion.

### Sample Processing and RNA Extraction

A venous sample was taken by venipuncture between 8 a.m. and 11 a.m. after 8 h of fasting, and the serum was immediately frozen at − 20 °C prior to testing. Adipose tissue biopsies were taken from abdominal subcutaneous fat and visceral fat at the beginning of the surgical procedure. Adipose tissue specimens were submerged in RNA stabilizing solution (RNAlater, Ambion) ^35^ and stored at − 80 °C until RNA extraction, which was performed using Trizol reagent (Invitrogen) according to the manufacturer’s protocol. RNA quantity and purity were determined by absorbance at 260 nm and 280 nm in a spectrophotometer. The integrity of the RNA was also examined with the Agilent 2100 Bioanalyzer system.

### Reverse Transcription and Real-Time Quantitative PCR

For mRNA expression analysis, complementary DNA (cDNA) was synthesized by reverse transcription using a commercial High Capacity cDNA Reverse Transcription Kit (Applied Biosystems) according to the manufacturer’s manual. Relative quantitative real-time polymerase chain reaction (qPCR) was performed using SYBR Green PCR master mix (Applied Biosystems) and gene-specific primer sets (Supplementary table 1). For reverse transcription of total RNA containing miRNA, a primer specific kit was used (miRCURY LNA Universal RT microRNA PCR, Exiqon), and miRNA expression was performed by qPCR using miRNA-specific primers (Supplementary table 1) and ExiLENT SYBR Green master mix, both from Exiqon. The qPCR experiments were performed in duplicate on a StepOnePlus™ Real-Time PCR System (Applied Biosystems), and primer specificity was verified by melt curve analysis. Threshold cycle (*C*_T_; number of cycles to reach threshold of detection) was determined for each reaction, and gene expression was quantified using the 2^−ΔΔCt^ method ^36^.

### Evaluation of Reference Genes for Normalization

To select suitable endogenous controls, the stability of expression of each potential reference gene was statistically analyzed with NormFinder ^37^, GeNorm [38], and BestKeeper ^39^ algorithms, which allow for the identification of the best reference genes for data normalization. For NormFinder and GeNorm, the GenEX (MultiD) software was used, and for BestKeeper, freely available Microsoft Excel-based software packages were employed ^40^. The selection of candidate endogenous controls for mRNA and for miRNA was performed separately, using two different sets of genes (Supplementary table 1). The candidate endogenous control genes for mRNA qPCR were actin, *GAPDH*, *36B4*, and *POLR2A*. For miRNA normalization, the candidate endogenous gene controls were miR-103a-3p, miR-24-3p, and U6.

### Adipokine and Hormone Assays

Multiplex Human Metabolic Hormone Magnetic Bead Panel (HMHEMAG-34 K) and Human Adipokine Magnetic Bead Panel (HADK1MAG-61 K) were used to analyze serum levels of insulin, C-peptide, glucagon, adiponectin, resistin, ghrelin, glucagon-like-peptide-1 (GLP-1), interleukin (IL)-6, and leptin. Analysis was performed according to the manufacturer’s instructions. Insulin resistance was estimated using the homeostasis model assessment of insulin resistance (HOMA-IR or HOMA model: fasting glucose * fasting insulin).

### Statistical Analysis

Quantitative variables were expressed as the mean (standard deviation [SD]), while qualitative variables were expressed as the absolute (*n*) and relative (%) frequencies. Differences among clinical and laboratory data between groups were assessed by the Mann–Whitney test (quantitative variables) and *χ*^2^ test (qualitative variables). Spearman’s rho (*ρ*) test was used to analyze the correlation between quantitative variables. Statistical analyses were performed using IBM SPSS Statistics version 25. A *P* value < 0.05 was regarded as significant.

### Bibliographic Search and Systematic Review

A systematic review of the literature was conducted in order to identify case–control studies that assessed mRNA expression of PPAR-γ in adipose tissue in obese patients compared to controls. All studies published before December 2020 were identified by searching the MedLine (source PubMed), Scopus, and Embase (which includes conference abstracts) databases, using the following entries as a search criterion in humans: “PPAR-γ mRNA expression,” “adipose tissue,” and “obesity.” The search was supplemented by reviewing references of original research reports and review articles on the topic. The language was restricted to English, and the search was supplemented using the MedLine option “Related Articles.” After the relevant studies were identified and selected, a standardized set of information was collected from each article including: the name of the authors, the year of publication, the journal of publication, the characteristics of the sample, the study design, the reference gene, and the result. This procedure was carried out according to the recommendations of the PRISMA Statement ^41^. Bibliographic search and data extraction were performed independently by three researchers (JLT, RUM, and IHG). Disagreements were resolved by consensus after consulting with another author (AJC).

## Results

### Analysis of the Basal Characteristics of Subjects Included

Our study included 43 obese subjects (34 women and 9 men) with a mean age of 44.9 (12.2) years old, a BMI of 49.3 (7.5) kg/m^2^, and a body fat percentage of 54.33 (3.83). In addition, 19 non-obese subjects (11 women and 8 men) were included with a mean age of 55.3 (17.4) years old, a BMI of 24.4 (2.1) kg/m^2^, and a body fat percentage of 31.46 (7.22). The basal characteristics of the subjects included in the study and the comparison between obese and non-obese subjects are summarized in Table 1. The only significant differences were that obese patients had higher BMI and body fat percentage and higher prevalence of obstructive sleep apnea–hypopnea syndrome.

**Table 1:** Basal characteristics of the obese and non-obese subjects

	Obese subjects *n* = 43	Non-obese subjects *n* = 19	*P*
Age (years)	44.9 (12.3)	55.3 (17.4)	0.054
Male (*n*)/Female (*n*)	9/34	8/11	0.085
BMI (kg/m^2^)	49.3 (7.5)	24.4 (2.1)	< 0.001
Hypertension, *n* (%)	18 (41.9)	6 (31.6)	0.444
Diabetes mellitus, *n* (%)	8 (18.6)	2 (10.5)	0.425
Cardiovascular disease, *n* (%)	2 (4.7)	0	0.339
Dyslipidemia, *n* (%)	12 (27.9)	3 (15.8)	0.304
Hypothyroidism, *n* (%)	8 (18.6)	2 (10.5)	0.425
OSAHS, *n* (%)	25 (58.1)	1 (5.2)	< 0.001
Prior cancer, *n* (%)	2 (4.7)	0	0.339
Fasting plasma glucose (mg/dL)	102.9 (32.1)	91.1 (12.4)	0.061
Total cholesterol (mg/dL)	197.4 (37.2)	197.9 (50.1)	0.880
Triglycerides (mg/dL)	134.9 (59.3)	120.6 (54.5)	0.551
LDL cholesterol (mg/dL)	117.5 (37.6)	123.8 (41.9)	0.733
HDL cholesterol (mg/dL)	48.1 (11.1)	50 (16.6)	0.985
Body fat percentage (%)	54.33 (3.83)	31.46 (7.22)	< 0.001

Quantitative variables are presented as mean (standard deviation), and qualitative variables are presented as *n* (%)

*BMI* body mass index, *LDL* low-density lipoprotein, *HDL* high-density lipoprotein, *OSAHS* Obstructive sleep apnea–hypopnea syndrome

### PPAR-γ1, PPAR-γ2, miR-27a, and miR-27b Expression in Subcutaneous and Visceral Adipose Tissue

**Fig. 1:**
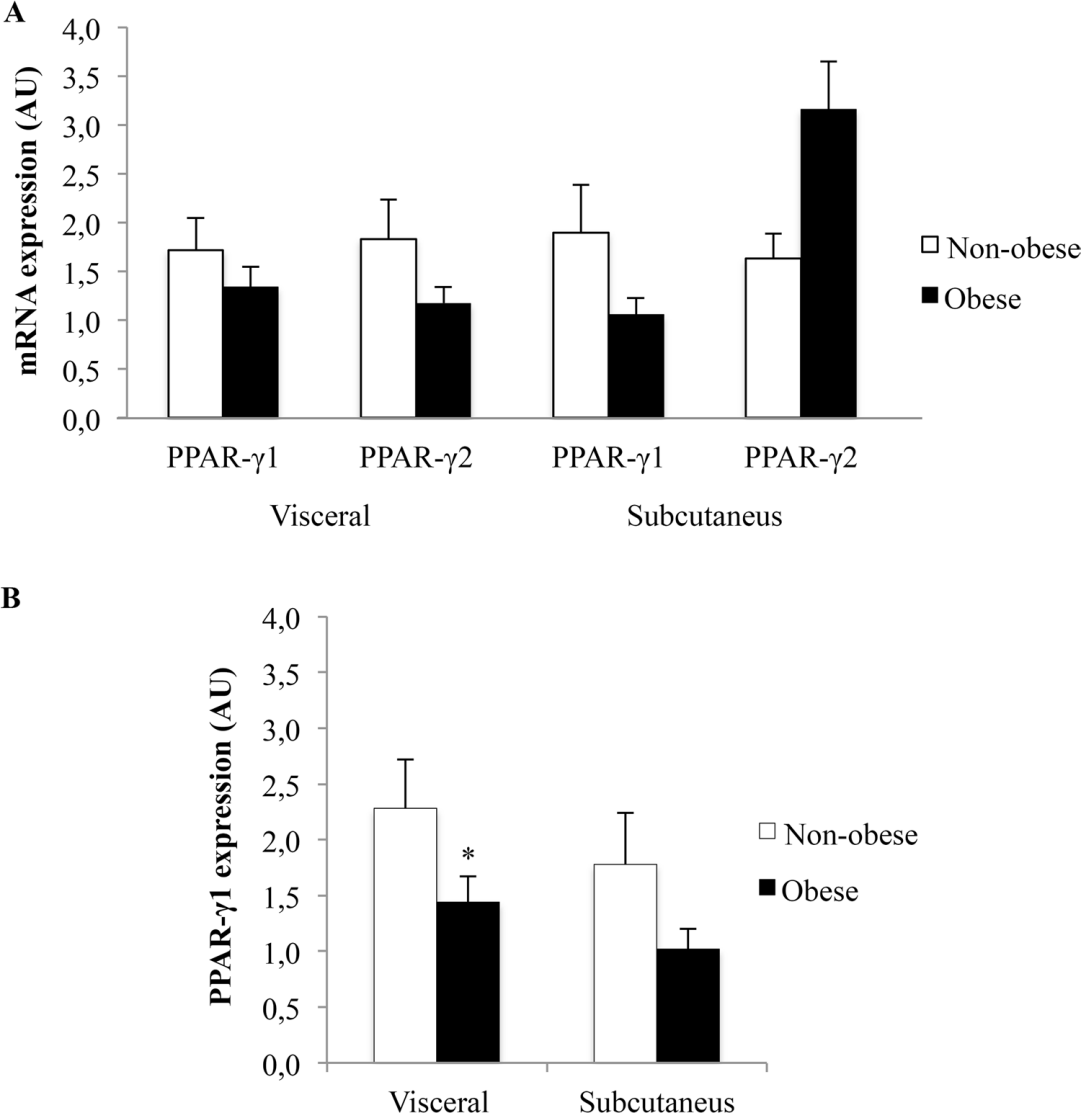
Relative mRNA expression in adipose tissue of obese patients and controls. **A**) PPAR-γ1 and PPAR-γ2 mRNA expression in visceral and subcutaneous adipose tissue in non-obese (*n* = 19) and obese (*n* = 43) subjects. **B**) PPAR-γ1 mRNA expression in non-obese women (*n* = 11) and obese women (*n* = 34). Results are expressed as a fold-change relative to the control group (AU: arbitrary unit; mean ± SEM; * *P* < 0.05)

The results of the evaluation of reference genes for normalization used in the analysis of mRNA expression in subcutaneous and visceral adipose tissue showed that actin and miR-103a-3p were the most stable reference genes (Supplementary Fig. 1). Thus, they were used for normalization of the expression of PPAR-γ1, PPAR-γ2, miR-27a, and miR-27b. Results regarding relative mRNA expression of PPAR-γ1 and PPAR-γ2 in subcutaneous and visceral adipose tissue of obese and non-obese subjects are shown in Fig. 1A. No significant differences were found in PPAR-γ1 and PPAR-γ2 mRNA expression in either tissue for the overall group. However, as shown in Fig. 1B, mRNA expression of PPAR-γ1 in visceral adipose tissue was significantly downregulated in obese women compared to non-obese women (fold increase = 1.43 vs. 2.27, respectively; *P* = 0.045). A non-significant downregulation was found for this comparison when we separately analyzed women 45 years or younger (fold increase = 1.32 vs. 3.04; *P* = 0.08) or women older than 45 years (fold increase = 1.54 vs. 1.84; *P* = 0.42). A non-significant trend was observed in subcutaneous adipose tissue (fold increase = 1.02 vs. 1.78; *P* = 0.084). PPAR-γ2 mRNA levels did not significantly differ between obese and non-obese women (data not shown), and the comparison was not performed in male obese patients due to the small sample size. In addition, the mRNA expression of PPAR-γ1 in visceral adipose tissue was negatively correlated with weight (*ρ* =  − 0.305, *P* = 0.016) and BMI, (*ρ* =  − 0.325, *P* = 0.001). There were no significant correlations between PPAR-γ1 in subcutaneous tissue and PPAR-γ2 in both adipose tissues with weight or BMI. Age was not significantly correlated with PPAR-γ1 or PPAR-γ2 expression.

**Fig. 2:**
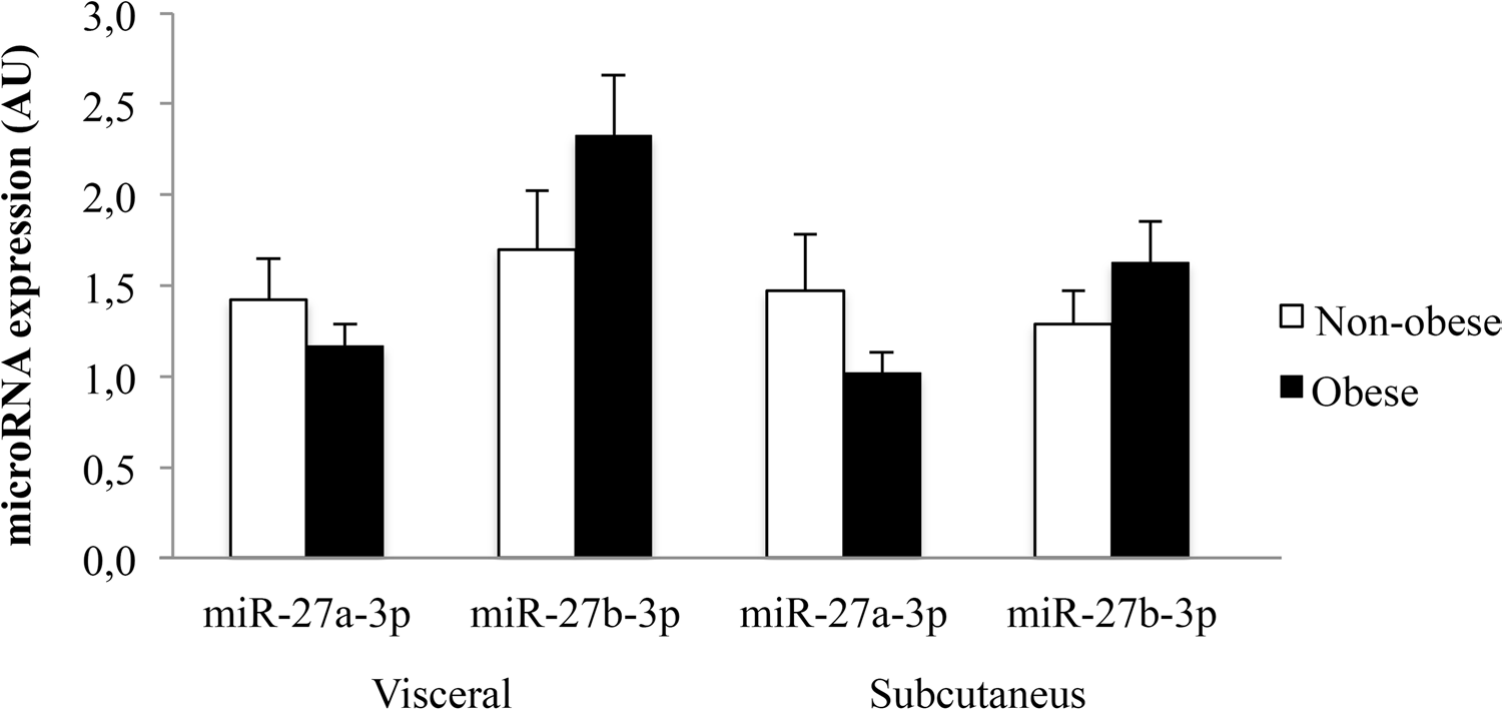
Relative microRNA expression in adipose tissue of obese patients and controls. miR-27a-3p and miR-27a-3p microRNA expression in visceral and subcutaneous adipose tissue in non-obese (*n* = 19) and obese (*n* = 43) subjects. Results are expressed as a fold-change relative to the control group (AU: arbitrary unit; mean ± SEM)

Due to the potential relationship between PPAR-γ and miR-27, we assessed the expression of miR-27a and miR-27b in subcutaneous and visceral adipose tissues, which did not differ between obese and non-obese subjects (Fig. 2). No sex-related differences were found in microRNA expression (data not shown). The results did not show any relationship between microRNA expression and anthropometric variables.

### Correlation Between PPAR-γ and miR-27 Gene Expression

The results showed a positive correlation between PPAR-γ1 and PPAR-γ2 expression in both adipose tissues (visceral adipose tissue: *ρ* = 0.398, *P* = 0.001; and subcutaneous adipose tissue: *ρ* = 0.286, *P* = 0.024). The results also showed a positive association between the expression of miR-27a and miR-27b in visceral adipose tissue (*ρ* = 0.399, *P* = 0.001). The expression of PPAR-γ1 and PPAR-γ2 did not significantly correlate with the expression of miR-27a and miR-27b.

### Association Between Gene Expression and Weight Loss After Surgery

**Fig. 3:**
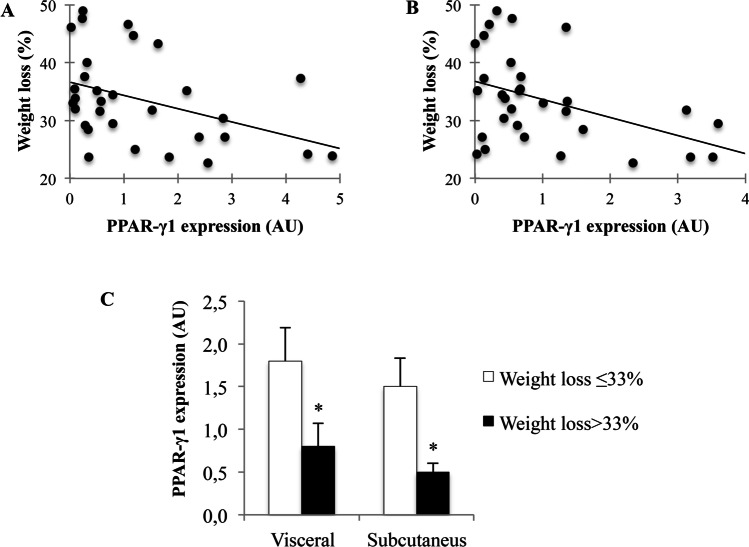
Relationship between baseline mRNA expression of PPAR-γ1 and weight loss after surgery. **A**) Correlation of mRNA expression in visceral adipose tissue and percentage of weight loss (*ρ* =  − 0.460; *P* = 0.009). **B**) Correlation of mRNA expression in subcutaneous adipose tissue and percentage of weight loss in subcutaneous adipose tissue (*ρ* =  − 0.404; *P* = 0.024). **C**) Relative mRNA expression of PPAR-γ1 in visceral and subcutaneous adipose tissue in patients who lost less than 33% of bodyweight (*n* = 15) and patients who lost more than 33% of bodyweight (*n* = 16). Results are expressed relative to values of the control group (AU: arbitrary unit; mean ± SEM; * *P* < 0.05)

Thirty-one patients (72%) were followed-up at least 1 year after surgery. The mean percentage of weight loss and percentage of BMI reduction were of 33.65% (7.7) and 33.64% (7.7), respectively. The relationship between the baseline PPAR-γ1, PPAR-γ2, miR-27a, and miR-27b expression, and weight loss and BMI reduction after surgery were analyzed. The results showed a significant negative correlation between the expression of PPAR-γ1 in subcutaneous and visceral adipose tissues, and the percentage of weight lost (in subcutaneous adipose tissue: *ρ* =  − 0.404, *P* = 0.024 and in visceral adipose tissue: *ρ* =  − 0.460, *P* = 0.009) (Fig. 3A, B) and percentage of BMI reduction (in subcutaneous adipose tissue: *ρ* =  − 0.398, *P* = 0.026 and in visceral adipose tissue: *ρ* =  − 0.462, *P* = 0.009). In addition, the patients who lost more than 33% of bodyweight had significantly decreased levels of PPAR-γ1 expression compared to patients who lost less than 33% of bodyweight (Fig. 3C). The results also showed a significant negative correlation between the expression of PPAR-γ1 in subcutaneous adipose tissues and the percentage of body fat loss (*ρ* =  − 0.367, *P* = 0.042). The expression of miR-27a in subcutaneous adipose tissue also was associated with the percentage of lost body fat (*ρ* = 0.407, *P* = 0.023). The remaining mRNA and microRNA gene expression data did not show any significant correlations with the decrease in weight and BMI after surgery. Among obese women, the results also showed a negative correlation between PPAR-γ1 expression in adipose tissue and the percentage of weight lost at 12 months (in subcutaneous adipose tissue: *ρ* =  − 0.423, *P* = 0.031 and in visceral adipose tissue: *ρ* =  − 0.490, *P* = 0.011) and percentage of BMI reduction at 12 months (in subcutaneous adipose tissue: *ρ* =  − 0.415, *P* = 0.035 and in visceral adipose tissue: *ρ* =  − 0.497, *P* = 0.01).

### Study of the Serum Adipokine and Hormone Levels

The relationship between the serum adipokine and hormone levels among obese and non-obese subjects is summarized in Table 2. These results showed that the serum levels of C-peptide, insulin, leptin, and HOMA-IR were higher in obese subjects than in non-obese (Table 2). Correlation analysis of the serum adipokine and hormone levels with gene expression in visceral and subcutaneous adipose tissue showed a significant direct correlation between the PPAR-γ1 expression in visceral adipose tissue and serum levels of GLP-1 (*ρ* = 0.358, *P* = 0.032). Among obese women, PPAR-γ1 expression in subcutaneous adipose tissue positively correlated with serum levels of insulin (*ρ* = 0.424, *P* = 0.017), leptin (*ρ* = 0.434, *P* = 0.01), and HOMA-IR (*ρ* = 0.410, *P* = 0.022), and PPAR-γ1 expression in visceral adipose tissue negatively correlated with adiponectin (*ρ* =  − 0.347, *P* = 0.044).

**Table 2:** Serum adipokine and hormone levels in obese and non-obese subjects

	Obese subjects *n* = 43	Non-obese subjects *n* = 19	*P*
C-peptide (pg/mL)	2366.18 (1205.27)	1495.23 (1331.39	0.001
Insulin (pg/mL)	996.79 (2875.66)	273.52 (383.82)	0.002
Glucagon (pg/mL)	9.22 (2.75)	8.1 (2.86)	0.056
GLP-1 (pg/mL)	23.41 (32.72)	69.93 (131.48)	0.620
IL-6 (pg/mL)	11.19 (13.41)	7.66 (3.82)	0.101
Ghrelin (pg/mL)	8.14 (3.78)	10.07 (15.76)	0.441
Leptin (ng/mL)	25.31 (12.19)	3.62 (4.04)	< 0.0001
Resistin (ng/mL)	163.67 (113.06)	152.02 (109.83)	0.663
Adiponectin (ng/mL)	312.93 (102.52)	339.7 (130.84)	0.094
HOMA-IR	2.98 (2.55)	1.16 (1.38)	0.003

Quantitative variables are presented as mean (standard deviation), and qualitative variables are presented as *n* (%)

*GLP-1* glucagon-like-peptide-1, *IL-6* interleukin-6

The correlation between serum adipokine and hormone levels with the rest of variables was also analyzed. Most relevant results showed that obese patients had a significant correlation between the leptin levels and the body fat percentage (*ρ* = 0.326, *P* = 0.037) and between HOMA-IR and serum levels of triglycerides (*ρ* = 0.408, *P* = 0.017) and HDL-cholesterol (*ρ* =  − 0.459, *P* = 0.006). Among obese women, a similar correlation of HOMA-IR with triglycerides and HDL-cholesterol was found. Of note, insulin levels in this subgroup were negatively correlated with percentage of total weight lost at 12 months (*ρ* =  − 0.424, *P* = 0.044) and percentage of BMI reduction at 12 months (*ρ* =  − 0.424, *P* = 0.044).

### Systematic Review of the Expression of PPAR-γ in Adipose Tissue

Among the published articles that analyzed adipose tissue mRNA expression of PPAR-γ gene in obese patients compared to controls, 24 studies were finally selected for inclusion (Supplementary Fig. 2). As shown in Table 3, three papers have analyzed the expression of PPAR-γ (PPAR-γ1, PPAR-γ2, or the common variant PPAR-γ) only in visceral adipose tissue, eleven in subcutaneous adipose tissue, and ten in both visceral and subcutaneous adipose tissue. To summarize, these papers have produced conflicting results: twelve previous studies have shown an increased mRNA expression of this transcription factor, eight studies have reported a decreased expression, and four studies have found no differences (Table 3). Of note, sample size in most studies is very small, especially regarding the number of controls. In addition, selection criteria of cases and controls differ between studies, with potentially relevant differences in variables such as age or BMI.

**Table 3:** Previous works analyzing expression of PPAR-γ gene in adipose tissue

First author, year	Reference genes	Targets genes	Obese (*N*)	Non-obese (N)	Principal results (compared to controls)
Lefebvre et al. 1997 ^14^	-	PPAR-γ1 PPAR-γ2	6	6	Increased expression of PPAR-γ in subcutaneous and visceral adipose tissue of obese patients
Auboeuf et al. 1997 ^23^	-	PPAR-γ1 PPAR-γ2	10	10	No differences in PPAR-γ1 and PPAR-γ2 expression in visceral adipose tissue
Vidal-Puig et al. 1997 ^17^	18S	PPAR-γ1 PPAR-γ2	24	14	Increased expression of PPAR-γ2 in subcutaneous adipose tissue of obese patients. Non-significant decreased expression of PPAR-γ1 in obese patients
Krempler et al. 2000 ^25^	GAPDH and actin	PPAR-γ	50/76*	19/20*	No differences in PPAR-γ expression
Redonnet et al. 2002 ^15^	Actin	PPAR-γ	15	10	Increased expression of PPAR-γ in subcutaneous adipose tissue of obese women
Sewter et al. 2002 ^60^	GAPDH	PPAR-γ1 PPAR-γ2	11	11	Increased expression of PPAR-γ1 and decreased expression of PPAR-γ2 in subcutaneous adipose tissue of obese patients
Bortolotto et al. 2007 ^12^	Actin	PPAR-γ	10	10	Increased expression of PPAR-γ in subcutaneous adipose tissue of obese patients, but no differences in visceral adipose tissue
Ceperuelo-Mallafre et al. 2007 ^27^	Actin	PPAR-γ	35	12	Increased expression of PPAR-γ in subcutaneous adipose tissue of obese patients, but no differences in visceral adipose tissue
Miranda et al. 2007 ^28^	Actin	PPAR-γ	22	24	No differences in expression of PPAR-γ in subcutaneous adipose tissue of obese patients
Poulain-Godefroy et al. 2008 ^21^	GAPDH	PPAR-γ1 PPAR-γ2	18	6	Decreased expression of PPAR-γ1 and PPAR-γ2 in subcutaneous adipose tissue of obese women, but not in visceral adipose tissue
Macias-Gonzalez et al. 2009 ^61^	18S	PPAR-γ1 PPAR-γ2	26	8	Decreased expression of PPAR-γ2 in visceral adipose tissue of obese patients compared with controls
Bairras et al. 2010 ^62^	Cyclophilin	PPAR-γ1 PPAR-γ2	32	12	Increased expression of PPAR-γ2 in visceral and subcutaneous adipose tissue of obese patients. No differences in expression of PPAR-γ1
Rodríguez-Acebes et al. 2010 ^22^	36B4	PPAR-γ1 PPAR-γ2	40	18	Decreased expression of PPAR-γ1 in subcutaneous adipose tissue of obese patients, but no differences in expression of PPAR-γ2
Ruschke et al. 2010 ^16^	18S	PPAR-γ2	95	58	Increased expression of PPAR-γ2 in visceral and subcutaneous adipose tissue of obese patients
Lee et al. 2010 ^13^	GAPDH	PPAR-γ	12	5	Increased expression of PPAR-γ subcutaneous adipose tissue of obese women
Kouidhi et al. 2011 ^18^	18S	PPAR-γ	7	6	Increased expression of PPAR-γ in obese in subcutaneous adipose tissue of obese patients
Hammes et al. 2012 ^24^	B2M	PPAR-γ	13	10	No differences in expression of PPAR-γ in subcutaneous and visceral adipose tissue
Leyvraz et al. 2012 ^19^	B2M	PPAR-γ1 PPAR-γ2	30	5	Decreased expression of PPAR-γ1 in subcutaneous and visceral adipose tissue of obese women, but no difference in expression of PPAR-γ2
Berhouma et al. 2013 ^11^	18S	PPAR-γ	262	83	Increased expression of PPAR-γ2 in subcutaneous adipose tissue of obese women
Moreno-Navarrete et al. 2013 ^20^	PPIA	PPAR-γ	25	31	Decreased expression of PPAR-γ in subcutaneous and visceral adipose tissue of obese patients
Núñez-Ruiz et al. 2016 ^63^	18S	PPAR-γ	15	11	Decreased expression of PPAR-γ in adipose tissue in obese subjects
Martínez-Jiménez et al. 2018 ^64^	GADPH	PPAR-γ	26	24	Increased expression of PPAR-γ in subcutaneous adipose tissue of obese subjects
Boughanem et al. 2019 ^65^	Actin	PPAR-γ2	23	15	Decreased expression of PPAR-γ2 in visceral adipose tissue of obese individuals
Tryggestad et al. 2019 ^66^	GAPDH	PPAR- γ	6	10	Decreased expression of PPAR-γ in subcutaneous adipose tissue of obese patients

^*^Sample size for subcutaneous/visceral fat samples

## Discussion

In our study, we have analyzed PPAR-γ and miR-27 gene expression in adipose tissue of obese patients, and we have not found clear differences between mRNA PPAR-γ1 or PPAR-γ2 expression in subcutaneous or visceral adipose tissue of obese patients compared with non-obese subjects, apart from a significant decrease only in women. We have also found rather discrepant results between previous studies analyzing this relationship. Among the several factors that may explain these conflicting findings, we should consider that an important number of variables may impact the analysis of PPAR-γ expression. Among them, we can include methodological variations such as the use of different reference genes. In this sense, multiple previous studies had used 18S gene which has been shown as one of the less stable reference genes in adipose tissue of obese patients ^42,43^. Other important variable which could impact PPAR-γ expression is the different characteristics between obese and non-obese subjects (e.g., age or gender or the type of adipose tissue analyzed), since PPAR-γ expression has been shown to differ in human adipocytes from subcutaneous superficial layer compared with deep layer adipocytes ^44^. Regrettably, between-study heterogeneity and small sample sizes make it difficult to analyze or confirm the role of any of these variables in PPAR-γ expression in adipose tissue as well as to compare with our own results. In our sample, age did not correlate with PPAR-γ expression although some previous data points towards a diminished PPAR-γ expression among older individuals ^45,46^.

It is of interest, however, that metabolic variables such as degree of insulin sensitivity have been previously associated to changes in miR-27 and PPAR-γ expression ^34,47^. Indeed, the development of peripheral insulin resistance and metabolic syndrome in some patients with obesity is related to the abnormal accumulation of triglycerides and other lipid species in non-adipose tissues (lipotoxicity) ^48^, which has been previously linked to decreased PPAR-γ expression in fat tissue ^34,48,49^. However, conflicting data from human and experimental studies correlating insulin resistance and PPAR-γ have been reported. First, several human and experimental studies point out that stimulation of the PPAR-γ receptor has positive effects on insulin resistance ^50,51^, suggesting that this receptor plays a protective role in lipotoxicity ^48,52^. Thus, an inverse correlation between PPAR-γ gene expression in adipose tissue and insulin resistance may exist ^25^, and it has also been described that miR-27a repression of PPAR-γ induces insulin resistance ^34,47,49^. Other authors, however, have found a direct correlation between PPAR-γ in adipose tissue and insulin resistance ^16^. The exact role of PPAR-γ in the pathophysiology of adipose tissue in obesity has yet to be elucidated, particularly because two similar studies with PPAR-γ adipose knockout mouse models also have conflicting results. In the study by Jones et al. ^53^, ablation of PPAR-γ in adipose tissue protected against the development of obesity and insulin resistance following a high fat diet. In contrast, another PPAR-γ knockout mouse model in adipose tissue has shown an increase in insulin resistance in adipose tissue and liver, but not in muscle tissue ^54^. These differences have been attributed to the point during development in which genetic recombination occurred that gave rise to the knockout, or to the recombination efficiency, but the findings remain difficult to interpret. In this scenario, we have found several significant correlations between PPAR-γ gene expression and adipokines and hormones, but our results did not show a consistent association across different tissues and groups between miR-27 or PPAR-γ gene expression and markers of insulin resistance. To summarize, although several data suggest that reduced PPAR-γ expression is directly correlated with insulin resistance, our data and results from previous studies do not allow us to reach definite conclusions. This situation along with the conflicting data regarding PPAR-γexpression in adipose tissue hampers the design of mechanistic experiments to address this association.

In this context, however, it is very interesting that we report for the first time that reduced expression of PPAR-γ1 is associated with more percentage of weight loss and BMI reduction at 1 year after sleeve gastrectomy. This result was consistent in both subcutaneous and visceral adipose tissue and also in the group of obese women (too few obese men were available for reliable statistical comparisons). In order to interpret this finding, we have to consider that sleeve gastrectomy has also a metabolic effect. Indeed, it has been reported that obese patients show improved glucose metabolism and significant hormonal changes after this technique ^55^, and mice who underwent this procedure, compared to those who underwent gastric bands, displayed increased adiponectin and GLP-1 levels, and improved insulin resistance and lipid profile ^56^. Therefore, we may hypothesize that patients with lower PPAR-γ1 expression may be more sensitive to metabolic changes after bariatric surgery, although further studies are needed to confirm this hypothesis. This hypothesis could be reinforced by the fact that, among obese women, basal fasting insulin serum levels, a marker of insulin resistance, were also negatively correlated with weight loss after surgery, and adiponectin levels, a marker of insulin sensitivity, were negatively correlated with PPAR-γ1 expression in visceral adipose tissue ^57,58^. Although we are well aware that the regulation of insulin resistance is far more complex, all our data together might point towards an association of certain markers of insulin sensitivity (reduced PPAR-γ1 expression and decreased insulin serum levels along with increased adiponectin levels) with more weight loss after bariatric surgery. In any case, our clinical data shows that lower PPAR-γ1 expression in adipose tissue could be a marker of better response to sleeve gastrectomy.

Regarding miR-27 gene expression in adipose tissue, we have not confirmed previous results by Viesti et al. ^59^, which showed a higher expression of miR-27a in visceral adipose tissue of obese patients compared to non-obese subjects. Nonetheless, we described that miR-27a expression in adipose tissue was positively correlated with the percentage of body fat loss after sleeve gastrectomy. Since it has been reported that miR-27a inhibits PPAR-γ gene expression ^34^, it would be tempting to speculate that higher levels of miR-27a expression could promote reduced PPAR-γ1 expression in adipose tissue and more weight loss after surgery, but more data are needed to support this hypothesis.

## Conclusion

In summary, obese patients might have changes in PPAR-γ expression in adipose tissue reflecting differences in insulin resistance and other variables such as sex, although these changes compared with controls are not clearly established according to our own results and the systematic review performed. In addition, we described that reduced PPAR-γ expression in adipose tissue is associated with increased weight loss after sleeve gastrectomy and could be a potential biomarker of better response after surgery.

## Supplementary Information

Below is the link to the electronic supplementary material.

Supplementary file1 (DOCX 71752 KB)
